# Caveats for Dispensers

**Published:** 1921-02-26

**Authors:** 


### CAVEATS FOR DISPENSERS.

### BY A CORRESPONDENT.

The loss in the medical profession of capacity to prescribe, in the sense of writing an original prescription in which each drug in the proper dose is separately specified, has long been regretted in medical journals. Such capacity to prescribe indicates some knowledge of dispensing, and therefore of the chemical compatibility of drugs. The factor which has so largely contributed to this loss is the compounding by wholesale chemists of endless therapeutic formulæ in tabloid form, designed to save the doctor's time in prescribing, and for the convenience of the patient in taking; also of fluid compounds of drugs in which the ingredients, with the dose, are given on the label.

### A Lost Art.

The growing effect of these methods during the last four decades has been to render prescribing in its former sense an almost lost art in the medical profession. Dispensing does not consist in merely putting prescribed quantities of drugs and chemical compounds into a medicine bottle. Knowledge of their properties—chemical and physiological—and of their chemical compatibility is indispensable to give the dispenser that skill and foresight to anticipate decompositions and their effects which is a *sine qua non* of qualification in compounding medicines. In addition to such knowledge, a sufficient sense of moral responsibility is also necessary.

Not infrequently have men possessing both, and holding the Pharmaceutical Society's certificate of qualification, been blamed and alarmed for some apparent mistake which they had not made. The following case at a leading shop in a fashionable town is an instance. The dispenser had sent out a mixture compounded from the prescription of a prominent physician, colourless. In a short time it was brought back as wrongly dispensed, being then a bright red. The dispenser was painfully alarmed, and said it was as much as his place was worth if his employer knew it. Soon, however, he found he had made no mistake. The mixture contained potass. iodid. and spt. ether nitrosi. It was colourless when first dispensed, but owing to there being always some free acid in the nitrous ether, in a short time it liberated the iodine from the iodide of potassium, rendering the mixture red.

A very risky mixture which many dispensers overlook is one in which the prescriber has ordered liquor hydrarg. perchlor. and spirit ammon. aromat. This, though generally sent out without, should always have a "Shake the bottle" label, and even then is unfit for internal use. The ammonia converts the perchloride of mercury into sal alembroth (white precipitate), a poisonous and insoluble salt. If there be no "Shake the bottle" label, the patient gets the whole in the last dose.

In a prescription I was once called to account for the doctor had ordered magnes. sulph. and ferri ammon. citratis. The mixture was, of course, clear, bright red when dispensed. In about a week some of it was brought back, clear pale green. The change was readily explicable; the sulphuric acid in the magnes. sulph. had gradually converted the ammonio-citrate of iron into sulphate of iron.

### An Important Point.

A very important point in dispensing is to shake spirit of chloroform, chloric ether, and tinct. chloroformi when ordered in watery mixtures, so as to dissolve the chloroform; otherwise it falls to the bottom in globules, and the patient gets it all in the last dose, burning the throat and stomach and almost stopping the breath. If other drugs in the prescription are such as would produce awkward froth with shaking, the spirit of chloroform must be shaken with half the bottle of water or infusion before they are added. Some dispensers are too wise to be taught on this important point, and will not dissolve the chloroform by shaking, but either send it out in globules at the bottom of the bottle, or strain it out so that the mixture does not contain it.

In case of a salt such as chlorate of potash being ordered in excess of its solubility in cold water, the only way is to send the surplus in the mixture in impalpable powder with a "Shake the bottle" label attached, as if hot water be used, on cooling, the chlorate beyond that soluble in cold water will crystallise out about the bottle.

Mixtures containing solution of strychnine or tincture of nux vomica, with sodium or potassium bicarbonate or such salts as potass. bromid. or potass. iodid., should have a "Shake the bottle" label attached.

These are a few of the more prominent and frequent, though often overlooked and therefore unanticipated, risks to which dispensers are liable.

The chemist to whom I was apprenticed was a perceptive, deliberative, and eminently cautious man, a favourable combination of qualities in a dispenser.

