# Caveats for Dispensers

**Published:** 1921-03-12

**Authors:** 


*Erratum.*

"Caveats for Dispensers."—*The Hospital*, p. 494, "Formulæ in tabloid form" in line 10 to read "Formulæ in *tablet* form."

